# Structural diversity of isoprene synthases in mosses from multiple terpenoid synthase lineages

**DOI:** 10.1016/j.jbc.2026.111410

**Published:** 2026-03-26

**Authors:** Tetsuya Kawakami, Sho Miyazaki, Yuya Inoue, Hiroshi Kawaide

**Affiliations:** 1 United Graduate School of Agricultural Science, Tokyo University of Agriculture and Technology (TUAT), Fuchu, Tokyo, Japan; 2 School of Science and Engineering, Tokyo Denki University, Hatoyama, Saitama, Japan; 3 Division of Land Plants, Department of Botany, National Museum of Nature and Science, Tsukuba, Ibaraki, Japan

**Keywords:** biosynthesis, bryophytes, diterpene, enzyme catalysis, enzyme mutation, isoprenoid, microbial type terpene synthase, terpenoid

## Abstract

Isoprene, a C5 hydrocarbon emitted by various land plants, serves as a protective molecule under heat stress. The moss *Calohypnum plumiforme* isoprene synthase (ISPS) catalyzes isoprene formation from dimethylallyl diphosphate and contains two aromatic residues (Y393 and F615) at the bottom of the active site that restrict the cavity size to accommodate the smaller C5 substrate. Here, we show that these residues are critical determinants of substrate size selectivity. Substitution of the two residues with alanine (Y393A and F615A) expanded the active site and exhibited diterpene synthase activity, generating *ent*-pimaradienes from *ent*-copalyl diphosphate. Further mutagenesis of the catalytic motif yielded *ent*-kaurene, demonstrating stepwise functional conversion from diterpene synthase to ISPS activity. Screening of ISPS genes across mosses identified two structurally unrelated enzyme classes: typical diterpene synthase–type ISPSs (*C*. *plumiforme*, *Pohlia nutans*) and microbial-type terpene synthase (TPS)–like ISPSs (*Polytrichum commune*, *Leucobryum juniperoideum*), the latter representing a previously unrecognized ISPS scaffold. Microbial-type TPS-like ISPSs of *P*. *commune*have two aromatic residues (W151 and F288), as in the case of *C*.*plumiforme*ISPS, to restrict the cavity size. Substitution experiments expanding the active site in ISPS of *P*. *commune* (W151A and F288A) determined the production of ocimenes as monoterpene synthase activity. These results define the molecular basis of substrate specificity in TPSs and demonstrate that structurally divergent enzyme families can independently acquire ISPS activity.

Isoprene (2-methyl-1,3-butadiene) is a volatile C5 terpene whose emission from plants has a significant impact on the atmospheric environment (1). Isoprene production has been confirmed across a broad range of land plant lineages, including angiosperms, gymnosperms, ferns, and bryophytes (mosses) (2, 3). In addition, isoprene production is induced by abiotic stresses, such as high temperature and intense light, and is thought to play a protective role by enhancing plant tolerance to these stresses (4, 5, 6).

In land plants, isoprene is enzymatically synthesized from C5 dimethylallyl diphosphate (DMADP) by isoprene synthase (ISPS), a member of the terpene synthase (TPS) family (7, 8, 9, 10, 11). Previous studies have primarily focused on ISPSs from angiosperms and have elucidated their evolutionary origins and structural basis (12, 13). Phylogenetic analysis has shown that angiosperm ISPSs are closely related to monoterpene (C10) synthases belonging to the TPS-b clade, a TPS subfamily specific to angiosperms. While ISPSs utilize DMADP as their substrate, monoterpene synthases generally use the C10 substrate geranyl diphosphate (GDP). The X-ray crystal structure of an ISPS from *Populus × canescens* was reported by Köksal *et al*. (14) in 2010, revealing that ISPSs possess two aromatic amino acid residues at the bottom of the active site, which restrict the active-site cavity and favor the smaller substrate DMADP. Mutational studies demonstrated that substitution of these aromatic residues with Ala converted an angiosperm ISPS into a monoterpene synthase, indicating that these residues are critical determinants of substrate size selectivity (15). In addition, it has been reported that almost all angiosperm ISPSs, except tomato ISPS (TPS47), are localized in plastids and utilize DMADP supplied *via* the 2*C*-methyl-D-erythritol 4-phosphate (MEP) pathway in plastids not *via* the mevalonate (MVA) pathway in the cytosol (16, 17, 18).

In gymnosperms, a hemiterpene alcohol (2-methyl-3-buten-2-ol C5) synthase from *P. sabiniana*has been reported to produce isoprene as a byproduct (19). Phylogenetically, 2-methyl-3-buten-2-ol C5 synthase from *P.sabiniana*belongs to the TPS-d clade, which is specific to gymnosperms, and is closely related to the monoterpene synthase lineage within the TPS-d clade (TPS-d1). These findings suggest that angiosperms and gymnosperms possess phylogenetically distinct ISPSs, respectively.

Given the phylogenetic diversity of ISPSs in seed plants, investigating the molecular mechanisms of ISPSs in mosses becomes essential to understand the structural and functional diversity of ISPS in land plants. Unlike seed plants, mosses possess unique terpene biosynthesis enzymes. The typical plant TPS family in mosses primarily consists of diterpene synthases belonging to the TPS-c clade, which is conserved across land plants. These include *ent*-copalyl diphosphate (*ent*-CDP) synthase (CPS) and bifunctional *ent*-kaurene synthase (CPS/KS), which are involved in the gibberellin biosynthetic pathway (20). In addition to these typical TPSs, mosses and other nonseed plants possess microbial-type terpene synthase–like (MTPSL) enzymes (21). MTPSLs are structurally different from typical plant TPSs: they consist of a single α-domain, whereas typical plant TPSs generally possess a β/α two-domain or γ/β/α three-domain structure. Furthermore, MTPSLs often contain variant forms of the highly conserved aspartate-rich motifs (such as DDxxxD instead of DDxxD in typical TPSs) that are essential for catalyzing the dephosphorylation and cyclization of prenyl diphosphate substrates. The presence of MTPSLs in bryophytes and ferns, but not in seed plants, suggests an ancient origin of these enzymes that may predate the divergence of vascular plants. However, the functional roles of most MTPSLs remain poorly characterized, and their potential contribution to specialized metabolism in nonseed plants is largely unexplored (21).

Our group previously identified and characterized an ISPS gene from the moss *Calohypnum plumiforme* (CpISPS) using genomic database resources combined with functional analyses (22). Phylogenetic analysis revealed that CpISPS belongs to the TPS-c clade, a group of diterpene synthases and clearly distinct from the monoterpene synthase clade that includes angiosperm and gymnosperm ISPSs. Structural modeling of CpISPS predicted the presence of two aromatic residues (Y393 and F615; Fig. 1*A*) at the bottom of the active site. The similarity of these residues to those found in angiosperm ISPSs led us to propose convergent evolution in C5 substrate recognition. However, the functional roles of these residues, the identity of a putative diterpene synthase ancestor, and the structural basis underlying functional conversion have not yet been experimentally validated. Moreover, although ISPSs appear to be broadly conserved in mosses, it remains unclear whether the structural features observed in CpISPS are representative of ISPSs across this lineage. In this study, we describe the structural and functional features of ISPSs identified from multiple moss species.

**Figure 1:**
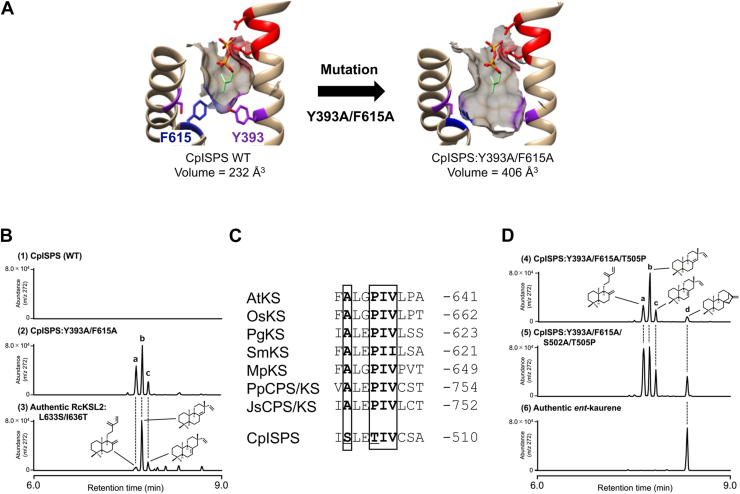
**Mutation experiments of CpISPS**. *A*, three-dimensional models of the active sites of WT and double mutant (CpISPS:Y393A/F615A). The structures predicted using AlphaFold2 were docked with DMADP, and the volumes of the active sites were calculated using POVME. The DDxxD motif is indicated in *red*. *B*, chromatograms from the GC–MS analysis of the products when CpISPS:Y393A/F615A and RcKSL2 double mutants reacted with *ent*-CDP. While CpISPS WT (1) showed no product, CpISPS:Y393A/F615A (2) showed three products (*A*–*C*) when reacted with *ent*-CDP. Product a was identified as *ent*-sclarene (*t*_R_ = 7.7 min), product b as *ent*-pimara-8(14),15-diene (*t*_R_ = 7.8 min), and product c as *ent*-pimara-7,15-diene (*t*_R_ = 7.9 min). The products from the RcKSL2 double mutant (3) were used to identify products (*A*–*C*) from the CpISPS mutant. The mass spectra of each product are shown in the supporting information (Fig. S1). *C*, multiple alignments of partial sequences of *ent*-kaurene synthases (KSs) from land plants and CpISPS. The AxxPIx motif highlighted in *squares* is conserved in land plant KSs but not in CpISPS. *D*, chromatograms from GC–MS analysis of CpISPS mutants in which the AxxPIx motif was introduced. Triple mutant (4), with a partially introduced motif, produced a small amount of *ent*-kaurene (d, *t*_R_ = 8.3 min). The quadruple mutant (5) with a fully restored motif showed an increased proportion of *ent*-kaurene. CpISPS, isoprene synthase gene from the moss *Calohypnum plumiforme*; DMADP, dimethylallyl diphosphate; POVME, Pocket Volume Measurer.

## Results

### Potential activity of *ent*-KS lies in CpISPS

**Figure 2:**
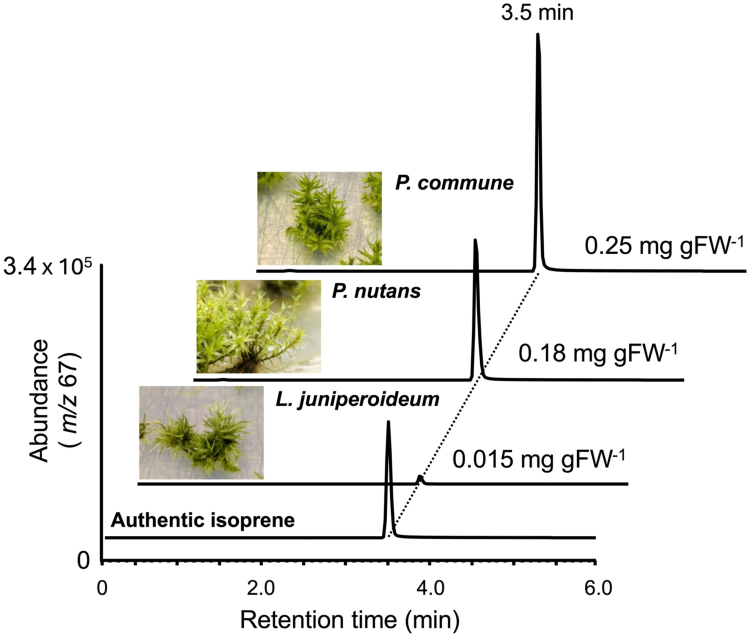
**Isoprene analysis of three mosses**. Chromatograms from GC–MS analysis of the headspace gases of three mosses: *Polytrichum commune*, *Pohlia nutans*, and *Leucobryum juniperoideum*. Isoprene production from mosses was confirmed by comparison with authentic isoprene, and the quantification of isoprene production to plant fresh weight (FW [mg/g]) was estimated based on each peak intensity.

The amino acid sequence of CpISPS shares 54% identity with the bifunctional *ent*-KS of *Physcomitrium* (*Physcomitrella*) *patens* (PpCPS/KS) within the TPS-c clade (23) and indicates a close evolutionary relationship despite their different substrate specificities. Three-dimensional modeling predicted that the active site of CpISPS was reduced by two aromatic residues (Y393 and F615), resulting in the restriction of a smaller substrate, such as DMADP (Fig. 1*A*). Replacement of these residues with small side-chain amino acids, such as Ala, was therefore expected to expand the active-site cavity and allow acceptance of larger substrates. Given that CpISPS belongs to the TPS-c clade, which predominantly comprises diterpene synthases, we hypothesized that expansion of the active site might restore diterpene synthase activity. To examine this possibility, we generated a double mutant, CpISPS:Y393A/F615A. Three-dimensional modeling using AlphaFold2 combined with POVME (Pocket Volume MEasurer) revealed that the predicted active-site cavity volume increased from 232 Å^3^ in wildtype CpISPS to 406 Å^3^ in the double mutant (Fig. 1*A*). This cavity size was comparable to those of other TPSs that accept C10–C20 substrates (Table S1). To evaluate the functional consequences of this expansion, we performed *in vitro* enzyme assays of the CpISPS:Y393A/F615A double mutant using a range of prenyl diphosphate substrates and analyzed the reaction products by GC–MS. When *ent*-CDP, a bicyclic C20 diterpene and the natural substrate of KSs, was supplied, the mutant converted *ent*-CDP into three products (Fig. 1*B*-2). These products were identified as diterpenes—*ent*-sclarene (peak a), *ent*-pimara-8(14),15-diene (peak b), and *ent*-pimara-7,15-diene (peak c)—based on direct comparison of retention times and mass spectra with those generated by RcKSL2:L633S/I636T (24) (Fig. S1). In addition to diterpene synthesis, the double mutant accepted shorter prenyl diphosphates, converting GDP and farnesyl diphosphate (FDP) into the acyclic monoterpenes myrcene/β-ocimene and the acyclic sesquiterpene β-farnesene, respectively (Fig. S2). In contrast, wildtype CpISPS did not accept GDP or FDP and produced no detectable products from these substrates. Notably, neither the wildtype enzyme nor the double mutant accepted geranylgeranyl diphosphate (GGDP). These results indicated that Y393 and F615 play critical roles in substrate size selectivity.

**Figure 3:**
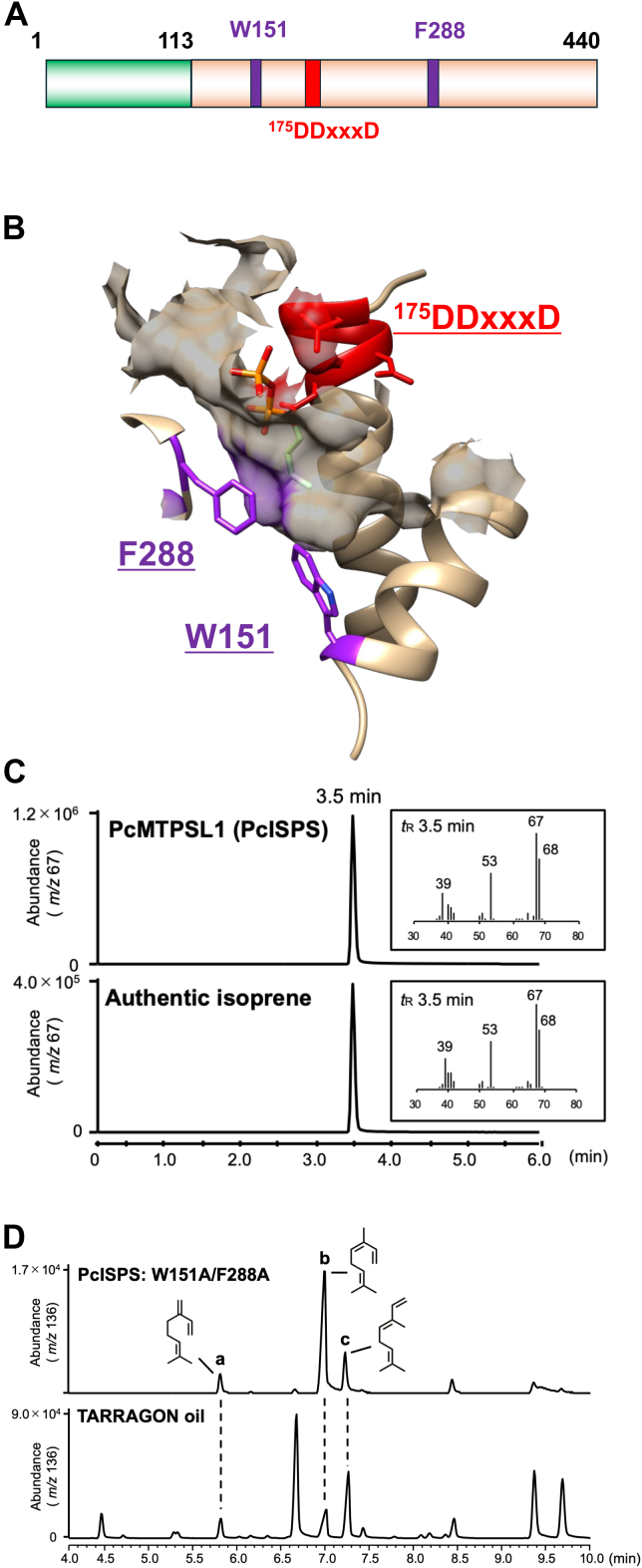
**The characterization of MTPSL-type ISPS in *Polytrichum commune***. *A*, the domain structure of PcMTPSL1 (PcISPS). It consists of 440 amino acids and comprises a putative chloroplast transit peptide (1–113 amino acid colored with *green*) and a catalytic domain (114–440 amino acids colored with *orange*). *B*, three-dimensional model of PcISPS docked with DMADP. The diphosphate group of DMADP was close to the DDxxxD motif of PcISPS shown in *red*, and the carbon chain was positioned inside the active site. W151 and F288 were positioned in the bottom of the active site to reduce the cavity size. *C*, the mass chromatograms of the *in vitro* ISPS assay that a recombinant PcISPS reacted with DMADP. *D*, the chromatogram from the GC–MS analysis of the product when PcISPS:W151A/F288A reacted with GPP, showing three products (*A*–*C*). Product a was identified as myrcene, product b as *cis*-β-ocimene, and product c as *trans*-β-ocimene. The chromatogram from a TARRAGON oil (Chemotyped Essential Oil; KENSO) was used for identification of products (*A*–*C*) from the PcISPS mutant. DMADP, dimethylallyl diphosphate; ISPS, isoprene synthase; MTPSL, microbial-type terpene synthase–like; PcISPS, ISPS gene from *Polytrichum commune*; PcMTPSL1, *P. commune* microbial-type terpene synthase–like 1.

**Figure 4:**
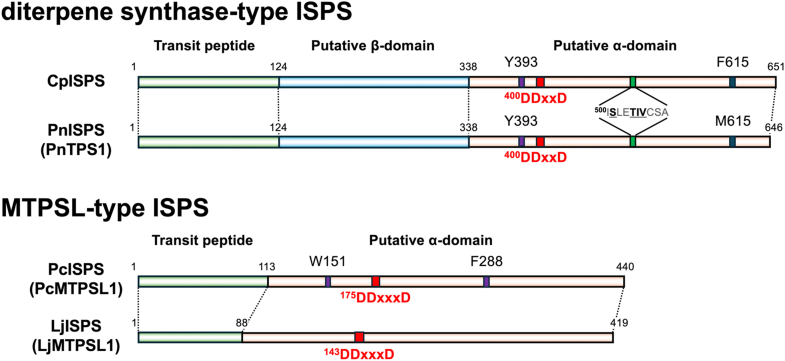
**The domain structures of diterpene synthase–type and MTPSL-type ISPSs in mosses**. The diterpene synthase–type ISPSs from *Calohypnum plumiforme* (CpISPS) and *Pohlia nutans* (PnISPS) comprise a putative chloroplast transit peptide (*green*) and two putative catalytic domains (β/α). In the α-domain of diterpene synthase–type ISPSs, the DDxxD motif, which is essential for a TPS activity involving dephosphorylation, is conserved. In addition, one or two aromatic residues (*purple*) were positioned at the bottom of the active site. The MTPSL-type ISPSs from *Polytrichum commune* (PcISPS) and *Leucobryum juniperoideum* (LjISPS) comprise the putative chloroplast transit peptide and a putative catalytic α-domain. In the α-domain of MTPSL-type ISPSs, the DDxxxD motif has a similar role to that of the DDxxD motif in canonical TPSs. In PcISPS, two aromatic residues (W151 and F288) are positioned at the bottom of the active site, whereas in LjISPS, no aromatic residue is positioned at the bottom of the active site. ISPS, isoprene synthase; MTPSL, microbial-type terpene synthase–like; TPS, terpene synthase.

**Figure 5:**
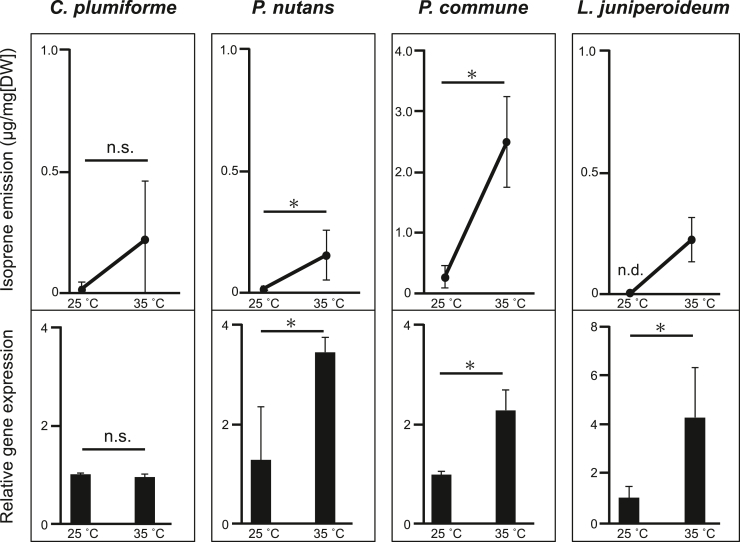
**Changes in isoprene production and ISPS gene expression of each moss under heat stress**. For the quantitative analysis of isoprene production, each gametophyte was incubated in a sealed vial for 6 h under continuous white light. Subsequently, the headspace gas was collected using SPME and subjected to GC–MS (nd, not detected; *n* = 3). For the quantitative analysis of ISPS gene expression, each gametophyte was incubated for 6 h under continuous *white light*. After that, each mRNA was extracted and subjected to quantitative RT–PCR based on the ΔΔCt method. The expression levels of ISPS genes were normalized to those of each housekeeping gene, as listed in Table S4. Three biological replicates were prepared for each treatment, and each sample was measured in duplicate. Statistical analysis was performed using Welch's *t* test (*∗p* < 0.05; ns, not significant; *n* = 3). The *asterisks* indicate significant differences. ISPS, isoprene synthase; SPME, solid-phase microextraction.

The production of *ent*-pimaradienes rather than *ent*-kaurene by the CpISPS:Y393A/F615A mutant suggests that KS activity requires additional structural features beyond simple expansion of the active-site cavity. Diterpene synthases forming labdane skeletons contain conserved amino acid motifs that determine the selective formation of tetracyclic *ent*-kaurene *versus* tricyclic *ent*-pimaradiene structures. In particular, the PIx motif in the F helix of KSs is responsible for *ent*-kaurene formation (25, 26). This motif is completely conserved in KSs but not in CpISPS (Fig. 1*C*). The absence of this motif in CpISPS likely explains why the CpISPS:Y393A/F615A mutant preferentially produces *ent*-pimaradienes instead of *ent*-kaurene. We hypothesized that the introduction of the KS-type motif into CpISPS might enable it to acquire KS activity. We therefore generated the triple mutant, CpISPS:Y393A/F615A/T505P. The enzyme assay with *ent*-CDP showed that this mutant produced three diterpenes similar to the product profile of CpISPS:Y393A/F615A, along with a small amount of *ent*-kaurene (Fig. 1*D*-4; peak d). Furthermore, an increase in *ent*-kaurene production was observed in the quadruple mutant CpISPS:Y393A/F615A/S502A/T505P (Fig. 1*D*-5). We next examined whether both an expanded active site and the KS-type motif (AxxPIx) are required for KS activity. CpISPS:S502A/T505P, which retains the AxxPIx motif but maintains a small active-site cavity, showed no activity toward *ent*-CDP (Fig. S3), indicating that this motif alone is insufficient without active-site expansion. These results demonstrated that the KS activity of CpISPS was acquired only when both the expansion of the active site and the AxxPIx motif were present.

### Isoprene production in various moss orders

Mosses are classified into 45 orders according to a recently published phylogenetic analysis (Fig. S4) (27). Two previously studied mosses, *C*. *plumiforme* and *P*. *patens*, belong to the orders Hypnales and Funariales, respectively. The former produces isoprene, whereas the latter does not (22). Taxonomically, they are very distant from each other. To investigate the isoprene production ability across diverse moss lineages, we screened mosses from a broad classification of orders for molecular analysis of isoprene biosynthesis and selected three species from different orders: *Pohlia nutans* (Bryales), *Polytrichum commune* (Polytrichales), and *Leucobryum juniperoideum* (Archidiales) (Fig. S4). Among them, *P*. *nutans* has a high-quality genome sequence database (28). The gametophyte of these three mosses was cultured at 25 °C for 24 h under white light (35 μmol m^–2^ s^–1^), and plant-emitted isoprene was collected with solid-phase microextraction (SPME) and analyzed by GC–MS. All three mosses produced isoprene, with remarkable production observed in *P*. *commune* (Fig. 2).

### Identification of the ISPS gene and enzyme function in *P*. *commune*

RNA-sequencing analysis was performed on total RNA extracted from sterilized gametophytes of *P*. *commune* grown under continuous light at 30 °C for 6 h to identify the ISPS gene. Given that CpISPS is phylogenetically related to the diterpene synthases in the TPS-c clade, we initially expected that the ISPS genes in *P*. *commune* would belong to the TPS-c clade. However, BLASTp searches against the *P*. *commune* transcriptome data revealed no homologs in the TPS-c clade. Instead, a hidden Markov model (HMM) search was performed using Pfam domains associated with TPSs (PF01397, PF03936, and PF19086) following published procedures (29, 30) to identify alternative candidate genes. This HMM search identified a single candidate gene encoding an MTPSL protein in *P*. *commune*. We designated this gene PcMTPSL1 (*P. commune* microbial-type terpene synthase-like 1). PcMTPSL1 encodes a protein of 440 amino acids with a transit peptide–like sequence at the N terminus, as predicted by three-dimensional modeling using AlphaFold2. The predicted structure of PcMTPSL1 consists of a single domain and the Asp-rich motif (DDxxxD), which is essential for catalyzing dephosphorylation (Fig. 3*A*). Interestingly, two aromatic amino acid residues (W151 and F288) were located at the bottom of the active site and likely reduce the cavity, similar to plant ISPSs (Fig. 3*B*).

The recombinant PcMTPSL1 protein (39 KDa) was produced in *Escherichia coli*, purified using Ni–NTA resin, and then subjected to an *in vitro* enzyme assay with DMADP as the substrate. GC–MS analysis showed that PcMTPSL1 catalyzed the conversion of DMADP into isoprene (Fig. 3*C*). When other prenyl diphosphates, GDP, FDP, and GGDP, were incubated with PcMTPSL1, no products were detected from any of these substrates (Fig. S5). Therefore, we identified PcMTPSL1 in *P*. *commune* (PcISPS) as a novel ISPS. As Y393 and F615 in CpISPS restrict the accommodation of larger substrates (Fig. S2), W151 and F288 in PcISPS were hypothesized to have a similar role. To test this hypothesis, we generated a PcISPS mutant in which these aromatic residues were replaced with Ala, PcISPS:W151A/F288A, and conducted enzyme assays incubated with prenyl diphosphates. When incubated with GDP, the mutant produced acyclic monoterpenes, such as myrcene and β-ocimene isomers (Fig. 3*D*), whereas no products were detected with FDP or GGDP (Fig. S6).

As described above, the presence of a putative transit peptide–like sequence at the N terminus of PcISPS prompted an *in silico* analysis of its subcellular localization. Prediction using WoLF PSORT suggested targeting of PcISPS to plastids (31). *In vivo* isoprene production in *P*. *commune* was strongly suppressed by fosmidomycin, an inhibitor of the plastidial MEP pathway. Application of fosmidomycin at concentrations ranging from 0.01 to 100 mM resulted in dose-dependent inhibition of isoprene emission, with complete inhibition observed at 100 mM (Fig. S7). Taken together, these results demonstrate that MTPSL-type PcISPS functions in a plastid-associated metabolic context by utilizing DMADP supplied from the MEP pathway.

### Identification of ISPS genes in *P*. *nutans* and *L*. *juniperoideum*

Phylogenetically, *P*. *nutans* and *L*. *juniperoideum* are distantly related to both *C*. *plumiforme* and *P*. *commune* (Fig. S4). Since the ISPS types in these two mosses were unknown, our objective was to determine whether they possess diterpene synthase–type or MTPSL-type ISPS. A genome sequence database was available for *P*. *nutans* (28), whereas no genomic or transcriptomic data were available for *L*. *juniperoideum*. Therefore, the ISPS genes from *P*. *nutans* (*PnISPS*) and *L*. *juniperoideum* (*LjISPS*) were identified using genome mining and RNA sequencing, respectively.

For *P*. *nutans*, four candidate TPS genes (GWHGBHNB028386, 031070, 032986, and 030381) were identified using a BLASTp search with CpISPS as the query. Among these, two genes (028386 and 032986) were successfully cloned from a complementary DNA (cDNA) library prepared from gametophytes of *P*. *nutans*. The predicted amino acid sequence of gene 028386, designated as PnTPS1, encoded a 645 amino acid polypeptide that showed 66% identity with CpISPS. The predicted structure of PnTPS1 consists of two domains (β/α) and possesses a DDxxD motif at the C-terminal region, the same as the structure of CpISPS (Fig. 4). Unlike CpISPS, however, the structure at the bottom of the active site of PnTPS1 lacks the two aromatic amino acid residues that are characteristic of ISPSs; instead, it contains one aromatic amino acid residue (Y393) and one Met residue (M615) (Fig. 4). Functional assays of recombinant PnTPS1 expressed in *E*. *coli* demonstrated ISPS activity (Fig. S8). PnTPS1 was therefore designated as the PnISPS, representing the second diterpene synthase–type ISPS identified in mosses. A cavity-expanding mutant of PnISPS (PnISPS:Y393A) was generated and subjected to functional analysis. GC–MS analysis of the products formed from *ent*-CDP by PnISPS:Y393A revealed the same product profile—*ent*-sclarene, *ent*-pimara-8(14),15-diene, and *ent*-pimara-7,15-diene—as observed for the CpISPS:Y393A/F615A mutant (Fig. S9). These results indicate that PnISPS, similar to CpISPS, acquires diterpene synthase activity upon active-site expansion. The other cloned gene, 032986 (PnTPS2). encodes a 789 amino acid without a predicted N-terminal transit peptide and is predicted to be a bifunctional CPS/KS-like diterpene synthase with a three-domain (γ/β/α) structure. Functional assay of recombinant PnTPS2 demonstrated that PnTPS2 functions as a bifunctional CPS/KS (PnCPS/KS) (Fig. S10).

For *L*. *juniperoideum*, two candidate ISPS-related genes were identified from RNA-sequencing data of isoprene-emitting gametophytes: an MTPSL-type gene (*LjMTPSL1*) and a bifunctional CPS/KS-like gene (*LjTPS1*). These candidates were obtained by HMM-based searches using Pfam domains associated with TPSs, following the same strategy used for PcISPS screening. LjMTPSL1 shares 36% amino acid sequence identity with PcISPS and contains an Asp-rich DDxxxD motif similar to that of PcISPS. In contrast, the two aromatic residues located at the bottom of the active site in PcISPS were not conserved in LjMTPSL1 (Fig. 4). LjMTPSL1 catalyzed the formation of isoprene from DMADP (Fig. S8). These results indicate that LjMTPSL1 functions as an ISPS (LjISPS). When other prenyl diphosphates—GDP, FDP, and GGDP—were incubated with LjISPS, no products were detected from any of these substrates (Fig. S5). The other TPS, LjTPS1, identified from the *L*. *juniperoideum* transcriptome, exhibited bifunctional CPS/KS activity (Fig. S10) and was designated as LjCPS/KS.

### Heat stress increases isoprene production and ISPS gene expression in mosses

The responses of isoprene emission and ISPS gene expression to heat stress were examined in four moss species (*C*. *plumiforme*, *P*. *nutans*, *P*. *commune*, and *L*. *juniperoideum*) to assess whether both increase and thus clarify the physiological role of isoprene production in mosses. All four moss species exhibited increased isoprene production (Fig. 5). *L*. *juniperoideum* did not produce detectable isoprene at 25 °C for 6 h, but substantial production was observed at 35 °C. In the other three moss species, isoprene production at 35 °C was approximately 10 times higher than at 25 °C. The relative expression levels of ISPS mRNA under high-temperature conditions were quantified by quantitative RT–PCR. As shown in Figure 5, ISPS expression increased approximately t–fourfold at 35 °C compared with 25 °C in all mosses except *C*. *plumiforme*.

## Discussion

Our previous study suggested that CpISPS might have evolved from diterpene synthases within the TPS-c subfamily clade. In angiosperms and gymnosperms, ISPS and monoterpene synthase show a close evolutionary relationship and are associated with the TPS-b and TPS-d subfamilies, respectively (12, 19). In 2017, Li *et al*. (15) experimentally demonstrated that a mutant enzyme with an expanded active site could be generated from an ISPS gene (*AdISPS*) isolated from the monocot *Arundo donax*. The mutant enzyme, AdISPS:F310A, switched to a monoterpene synthase activity and produced acyclic monoterpenes such as β-ocimene, whereas the wildtype enzyme did not produce any monoterpenes. Analogous to the experimentally supported acyclic monoterpene synthase origin of angiosperm ISPSs, the hypothesis that CpISPS originated from diterpene synthases needs to be experimentally verified. The cavity of the active site in CpISPS was expanded by replacing both aromatic amino acids with Ala, resulting in diterpene cyclase activity producing *ent*-pimaradiene (Fig. 1*B*). A notable difference between AdISPS:F310A and CpISPS:Y393A/F615A was that the latter acquired a cyclization activity solely through cavity-expanding mutations. Previous studies have demonstrated that land plant KSs can be converted into diterpene cyclases involved in secondary metabolism through minimal amino acid substitutions (20, 24, 25). In particular, a single mutation in the PIx motif of KSs converts KS activity to *ent*-pimaradiene synthase activity (25). Focusing on the PIx motif and the upstream alanine residue (AxxPIx) conserved among land plant KSs (Fig. 1*C*), we constructed a quadruple mutant, CpISPS:Y393A/F615A/S502A/T505P, which exhibited *ent*-KS activity (Fig. 1*D*). These mutational analyses suggested that KS retains substantial catalytic plasticity, enabling functional diversification into other diterpene synthases as well as ISPSs.

We identified another diterpene synthase–type ISPS (PnISPS) in *P*. *nutans*, a moss distantly related to *C*. *plumiforme* (Fig. S4). Although PnISPS shares an aromatic residue (Y393) at the bottom of the active site with CpISPS, the position corresponding to F615 in CpISPS is occupied by Met (M615) in PnISPS (Fig. 4). Nevertheless, the estimated active-site volume of PnISPS was similar to that of CpISPS (Table S1). In CPS/KS (PpCPS/KS) in *P*. *patens*, substitution of A610 with Met or Phe inhibited 16α-hydroxy-*ent*-kaurane production and led to *ent*-kaurene as the single product (32). Both Met and Phe possess bulky and highly lipophilic side chains and likely play the same role in reducing active-site volume. The cavity-expanded mutant PnISPS (PnISPS:Y393A) produced *ent*-pimaradiene isomers (Fig. S9), and the amino acid sequences of the AxxPIx motifs in PnISPS and CpISPS were identical (Fig. 4). These experimental results from two moss ISPSs suggested that diterpene synthase–type ISPSs evolved from KS *via ent*-pimaradiene synthase activity and subsequently to ISPS. Furthermore, *C*. *plumiforme* and *P*. *nutans* were phylogenetically distant from each other, suggesting the possibility that diterpene synthase–type ISPSs are widely distributed among moss species.

**Figure 6:**
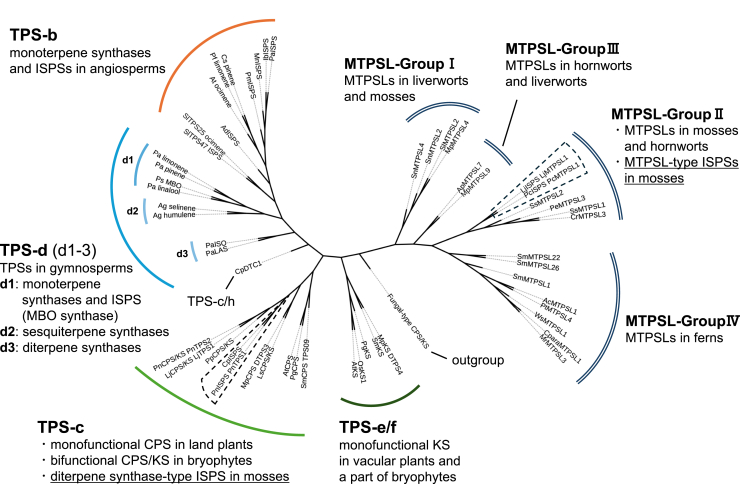
**Phylogenetic tree analysis of TPSs and MTPSLs in terrestrial land plants**. Enzyme genes that have been functionally characterized in previous studies were used (Table S6). The ISPSs from mosses are highlighted by *dotted lines*. ISPS, isoprene synthase; MTPSL, microbial-type terpene synthase–like; TPS, terpene synthase.

We initially hypothesized that diterpene synthase–type ISPSs are the primary ISPSs conserved in mosses. Unexpectedly, our search revealed previously unrecognized diversity in ISPS structures, including the identification of MTPSL-type ISPSs in *P*. *commune* and *L*. *juniperoideum*, which are structurally and phylogenetically distinct from the diterpene synthase type. Nonseed plants broadly possess MTPSL-encoding genes that are absent in seed plants (33). Originally identified through a genome-wide analysis of the fern *Selaginella moellendorffii* (29), MTPSL genes have been reported across diverse nonseed plant lineages, including ferns, mosses, liverworts, and hornworts (34, 35, 36, 37, 38). Previous studies reported that MTPSLs utilize GDP or FDP as substrates to produce monoterpenes and sesquiterpenes, respectively (21). Despite belonging to the MTPSL family, PcISPS and LjISPS differ from previously characterized MTPSLs in that they recognize only DMADP as their substrate (Fig. S5). A previous study classified MTPSLs in nonseed plants into four subfamilies (group I–IV) (33). Phylogenetic analysis placed PcISPS and LjISPS within the group II MTPSL subfamily (Fig. 6). Group II MTPSL homologs are widely distributed among mosses (33), suggesting that MTPSL-type ISPSs may be as broadly distributed in moss species as diterpene synthase–type ISPS.

Three-dimensional modeling of PcISPS predicted that W151 and F288 are located at the bottom of the active site, thereby restricting substrate accommodation (Fig. 3*B*). A cavity-expanded mutant (PcISPS:W151A/F288A) converted GDP into acyclic monoterpenes (Fig. 3*D*), indicating that these aromatic residues at the active-site bottom play a role analogous to those in angiosperm ISPSs and diterpene synthase–type ISPSs. However, because LjISPS naturally lacks aromatic residues at the bottom of the active site (Fig. 4), the substrate recognition mechanisms in ISPSs likely involve additional structural determinants beyond the active-site bottom architecture alone. Isoprene biosynthesis in plants occurs in plastids and is strongly inhibited by fosmidomycin, an MEP pathway inhibitor (17). Fosmidomycin treatment significantly reduced isoprene production in *P*. *commune* (Fig. S7), indicating that PcISPS utilizes DMADP supplied by the plastidial MEP pathway, the same as angiosperm ISPSs (16, 17, 18). Together, these results suggest that, whereas MTPSLs generally exhibit broad substrate specificity, PcISPS and LjISPS constitute a specialized subgroup adapted for isoprene biosynthesis *via* the MEP pathway in mosses, reflecting an alternative route for the acquisition of ISPS function within the MTPSL family.

Previous studies have suggested that isoprene emission in seed plants contributes to the reduction of cellular damage caused by abiotic stresses, such as heat and oxidative stress (4, 5, 6). In angiosperms, ISPS genes would increase in response to the abiotic stresses. In mosses, the isoprene emission increased by heat stress (3), but there was no knowledge that the expression of the ISPS gene in mosses also increased with stresses. In this study, we confirmed that all moss species, except *C*. *plumiforme*, exhibited increased isoprene emissions and elevated ISPS gene expressions under high temperatures similar to angiosperm ISPSs (Fig. 5). Although moss species exhibit distinct ecological adaptations, further studies are needed to identify the environmental factors that impose stress or confer tolerance in *C*. *plumiforme*.

These results demonstrate that mosses possess multiple types of ISPSs, each defined by distinct molecular bases underlying enzyme structure and substrate recognition, as revealed through comparisons with angiosperm ISPSs. This diversity in early land plants provides a framework for future studies aimed at elucidating the evolutionary relationships and diversification of ISPSs across moss lineages.

## Experimental procedures

### Materials

Moss plants used in this study were collected and cultivated as follows. *P*. *commune* was purchased from Mossfarm, Inc., and *L*. *juniperoideum* was collected from Kuroyama-Santaki. These two mosses were identified by the authors. The moss *P*. *nutans* was provided by Dr Hiroyasu Motose (Okayama University). The gametophytes of *C*. *plumiforme*, *P*. *commune*, and *L*. *juniperoideum* were cultured on BCDAT agar medium at 25 °C under an 8 h dark/16 h light cycle, as described in a previous article (22). In the case of *P*. *nutans*, the gametophytes were cultured on 10% MS agar medium at 25 °C under an 8 h dark/16 h light cycle.

### GC–MS analysis of isoprene emission and quantification from gametophytes of mosses

For isoprene qualitative analysis in four mosses, 0.1*g* fresh weight (FW) of gametophytes was placed into a 50 ml glass vial (40 × 75 mm; Nippon Electric Glass, Co, Ltd) with a septum lid (Nippon Electric Glass) and incubated at 30 °C for 24 h under continuous white light. After the treatment, the sample was placed into darkness to suppress isoprene biosynthesis until GC–MS analysis. Isoprene gas in the vial was collected using SPME (fiber holder and fiber assembly: 75 μm carboxen/polydimethylsiloxane, Supelco, Merck). The glass vial was placed on the hot plate at 40 °C to volatilize isoprene, the needle of SPME was inserted in the vial, and the SPME fiber was exposed to the headspace for 5 min and then subjected to GC–MS. The GC–MS system (GC 6890 Series Plus and MSD 5975 Series; Agilent Technologies) for isoprene was coupled with a DB-624 column (30 m long, 0.25 mm internal diameter, 1.40 μm film thickness; Agilent J&W). The flow rate of the He carrier gas was 1.22 ml/min, the injector temperature was 160 °C, and the injection was performed in splitless mode. The column oven was kept at 40 °C for 6 min, and the samples were injected *via* SPME, according to the manufacturer’s protocol. For isoprene quantitative analysis, moss gametophytes (0.1*g* FW) were placed into a 50 ml vial sealed with a septum lid and incubated under continuous white light conditions at 25 or 35 °C for 6 h. After incubation, the headspace gas was collected using SPME and subjected to GC–MS analysis using the system for isoprene, as mentioned above.

### RNA-sequencing analysis of *P*. *commune* and *L*. *juniperoideum*

For transcriptome assembly and gene identification, RNA sequencing was performed. Gametophytes of *P*. *commune* and *L*. *juniperoideum* grown on agar medium were pretreated in darkness at 25 °C for 1 day. After a darkness treatment, 0.1*g* FW of gametophytes was placed into a 50 ml glass vial with a septum lid and incubated at 30 °C for 6 h under continuous white light. After the 6 h treatment, the gametophytes were frozen in liquid nitrogen, and total RNA was extracted from the gametophytes using PureLink Plant RNA Reagent (Thermo Fisher Scientific) according to the manufacturer's protocol. The RNA concentration and quality were determined using an Agilent Bioanalyzer 2100 (Agilent Technologies). RNA sequencing was conducted by DNAFORM Co, Ltd, and a total of approximately 120 million paired-end reads were acquired from each moss. Raw reads were trimmed using Trim Galore! (version 0.6.7), and the processed reads were assembled using Trinity (version 2.13.2).

### Gene cloning of candidate enzymes

For cloning the candidate genes, each cDNA library was prepared from total RNA using SuperScript IV Reverse Transcriptase (Thermo Fisher Scientific). To clone the candidate cDNAs of PnTPS1-2, PcMTPSL1, LjMTPSL1, and LjTPS1 from each cDNA library, nested-PCR was performed using KOD-Plus-Neo (TOYOBO) with primers listed in Table S2. First-PCR was performed to amplify the candidate cDNAs from each cDNA library. Second-PCR was performed to amplify cDNA fragments, excluding the chloroplast transit peptide and the stop codon and adding homologous regions required for the SLiCE reaction (39). The amplified cDNA fragments were cloned into pET21a(+) (Sigma–Aldrich) or pColdⅡ (Takara Bio) *via* the SLiCE reaction, and the nucleotide sequences were confirmed through DNA sequencing. To confirm an authentic sequence of PnTPS1 (PnISPS) identified in the genome database, the full-length cDNA of PnISPS was also cloned into pET21a(+) and was subjected to DNA sequencing.

### Mutant construction

Site-directed mutagenesis of CpISPS, PnISPS, and PcMTPSL1 (PcISPS) was conducted *via* inverse-PCR. Inverse-PCR was performed using KOD-Plus-Neo with primers listed in Table S2. The blunt ends of the amplified fragments were phosphorylated using T4 Polynucleotide Kinase (Nippon Gene), and the fragments were self-ligated using Ligation High, version 2 (TOYOBO). The mutant sequences were confirmed through DNA sequencing.

### Recombinant protein expression in *E*. *coli*

Recombinant proteins of CpISPS mutants, PnISPS and its mutants, PnTPS2 (PnCPS/KS), PcISPS, LjMTPSL1 (LjISPS), and LjTPS1 (LjCPS/KS) were expressed in *E*. *coli* and purified as previously described (22). The constructs cloned from the cDNAs into pET21a(+) or pColdⅡ were transformed into *E*. *coli* strain BL21 (DE3) pLysS (Thermo Fisher Scientific). The transformed *E*. *coli*cells were precultured for 16 h at 37 °C in 2 ml of LB medium containing 100 μg/ml ampicillin and then cultured at 37 °C in 200 ml of LB medium containing ampicillin until the cell density reached 0.6 at an absorbance at 600 nm. Recombinant protein expression was induced by adding IPTG (0.1 mM), and the cultures were incubated at 18 or 16 °C for 16 h. Cells were collected by centrifugation at 2100*g* for 15 min and resuspended in 5 ml of buffer A (100 mM Tris–HCl [pH 7.5] and 10% [v/v] glycerol). The cells were disrupted by treatment with lysozyme (final concentration: 1.5 mg/ml), followed by sonication using Sonic Tower UDS-200 (TOMY SEIKO Co, Ltd) at 100 W (10 s on, 10 s off, 6 cycles), and the soluble fraction was obtained by centrifugation at 10,600*g* for 30 min. Imidazole and NaCl were added to the soluble fraction to final concentrations of 10 mM and 300 mM, respectively, and the mixture was applied to a Ni–NTA agarose resin (FUJIFILM Wako Pure Chemical Corporation) packed in an 8 ml empty column. The resin was washed with 20 ml of wash buffer (50 mM Tris–HCl [pH 8.0], 300 mM NaCl, and 20 mM imidazole), and the His-tagged recombinant protein was eluted with 2 ml of elution buffer (50 mM Tris–HCl [pH 8.0], 300 mM NaCl, and 250 mM imidazole). Protein concentrations were determined using the Bradford protein assay with bovine serum albumin (0.1–1.0 mg) as a standard.

### Enzyme assay for ISPS activity

For functional analysis of ISPS activity, an *in vitro* enzyme assay using an enzyme cocktail was conducted as previously described (22). The recombinant proteins were mixed with a basic enzyme cocktail that provides DMADP (a reconstruction of the MVA pathway starting with MVA) in a 2 ml glass vial with a septum lid. After incubation at 38 °C for 1 h, the headspace gas was collected using SPME and subjected to GC–MS analysis. The GC–MS analysis method was as mentioned above.

### Enzyme assay for diterpene synthase activity

To confirm the enzyme activity of diterpene synthase, the recombinant proteins were reacted with GGDP or *ent*-CDP using the enzyme cocktail. GGDP synthase from *Neurospora crassa* and *ent*-CDP synthase from *Oryza sativa* (OsCPS1) were used for producing GGDP and *ent*-CDP when mixed with the basic enzyme cocktail (40, 41). An enzyme mixture (500 μl scale) containing the target proteins, GGDP synthase from *N*. *crassa*, OsCPS1, and the basic enzyme cocktail started a reaction adding MVA and incubated at 30 °C for 16 h. After incubation, the enzyme products were extracted three times with an equal volume of hexane. The extract was concentrated using a centrifugal evaporator (miVac Sample Concentrator; ATS Genevac, PA, USA) and resuspended in 50 μl of hexane, and then 1 μl of the extract was subjected to GC–MS analysis. The GC–MS system (Agilent Technologies; GC 6890 Series Plus and MSD 5975 Series) for diterpenes was coupled with a DB-1 column (Agilent J&W; 15 m long, 0.25 mm internal diameter, 0.25 μm film thickness). The flow rate of the He carrier gas was 1.0 ml/min, the injector temperature was 250 °C, and the injection was performed in splitless mode. The column oven was kept at 80 °C for 1 min, then heated at 20 °C/min to 200 °C and at 5 °C/min to 250 °C.

### Enzyme assay for monoterpene and sesquiterpene synthase activity

To confirm the activity of monoterpene and sesquiterpene synthase, the recombinant proteins were reacted with GDP or FDP using an enzyme cocktail (40, 41). GDP synthase from *O*. *sativa* (OsGPS1) and FDP synthase from *N*. *crassa* (NcFPS) were used for producing GDP and FDP when mixed with the basic enzyme cocktail. A 500 μl mixture containing the target proteins, the basic enzyme cocktail, and OsGPS1 or NcFPS started a reaction by a adding MVA in a 2 ml vial sealed with septum lid and incubated for 3 h at 30 °C. After incubation, the headspace gas was collected using SPME and subjected to GC–MS analysis. The GC–MS system for monoterpenes and sesquiterpenes was coupled with a DB-1 column (15 m long, 0.25 mm internal diameter, 0.25 μm film thickness; Agilent J&W). The flow rate of the He carrier gas was 1.0 ml/min, the injector temperature was 250 °C, and the injection was performed in splitless mode. The column oven was kept at 40 °C for 1 min, then heated at 5 °C/min to 80 °C, held for 3 min, and then at 30 °C/min to 240 °C.

### Three-dimensional modeling of the active sites of ISPSs

Three-dimensional modeling was conducted following a method as previously described (22). The three-dimensional structures of ISPSs were predicted using AlphaFold2. Molecular docking simulations of these enzymes with their substrates (DMADP or *ent*-CDP) were conducted using AutoDock Vina (The Scripps Research Institute). The predicted structures of enzymes docked with their substrates were visualized using UCSF Chimera (version 1.16). Calculation of the volume of the predicted substrate binding cavities was performed using POVME 3.0 (University of California) (42).

### Inhibitor assay for isoprene synthesis

Fosmidomycin (Fujisawa Pharmaceutical Co, Ltd) was dissolved in water at 10 μM, 1 mM, and 100 mM. Gametophytes of 0.1*g* FW of *P*. *commune* were treated with the solutions and then incubated in darkness at 25 °C for 24 h. After incubation, these gametophytes were placed into a 50 ml glass vial sealed with a septum lid and incubated under continuous white light at 25 °C for 6 h. After the treatment, the headspace gas was collected and subjected to GC–MS analysis.

### Quantitative real-time PCR

The 0.1*g* FW of gametophytes of each moss species was incubated under continuous white light for 6 h at either 25 or 35 °C. After incubation, the samples were immediately frozen in liquid nitrogen and stored at −80 °C until subsequent experiments were performed. Total RNA was extracted from the frozen samples using RNAiso Plus (Takara Bio) and reverse-transcribed into cDNA using ReverTra Ace qPCR RT Master Mix with gDNA Remover (TOYOBO) following the manufacturer's protocols. For the quantitative RT–PCR experiment, each target was amplified using THUNDERBIRD Next SYBR qPCR Mix (TOYOBO) and detected using a QuantStudio 1 Real-Time PCR System (Applied Biosystems). The homologs of the housekeeping genes encoding β-actin or α-tubulin were used as reference genes (Table S4). Specific primers for the ISPS genes and the reference genes are listed in Table S5. Three biological replicates of each treatment were prepared, and each biological replicate was measured in technical duplicate. Relative expression levels of the ISPS genes were calculated using the 2^–ΔΔCt^ method.

### Phylogenetic tree analysis

To construct the phylogenetic tree of TPSs and MTPSLs, genes encoding functionally characterized enzymes were used as shown in Table S6. The amino acid sequences were aligned using MAFFT, version 7.511, with the L-INS-i method *via* the website. A maximum-likelihood tree was constructed using IQ-TREE, version 2.3.6, with the Q.pfam+F+R3 model, which was determined by ModelFinder Plus implemented in the software based on the Bayesian information criterion. The bootstrap values were calculated from 1000 replicates. The tree was visualized in FigTree, version 1.4.4. In this study, we illustrated the tree unrooted because the evolutionary origin between TPSs and MTPSLs was unclear.

### Statistical analysis

All experiments were performed with three biological replicates unless otherwise stated. Statistical analyses were conducted using R. For the fosmidomycin treatment experiment, one-way ANOVA followed by the Tukey–Kramer post hoc test was performed (*n* = 3). Significant differences among groups are indicated by different letters (*p* < 0.01). For heat stress experiments (isoprene production and ISPS gene expression), Welch's *t* test was applied (*n* = 3), with asterisks indicating significant differences (∗*p* < 0.05, ∗∗*p* < 0.01). Data are presented as mean ± SD.

## Data availability

The characterized enzyme genes in this study were available in the DNA Data Bank of Japan database under the following accession numbers: PnISPS, LC898145; PcISPS, LC898146; LjISPS, LC898147; PnCPS/KS, LC898148; and LjCPS/KS, LC898149. The transcriptome data of *P*. *commune* and *L*. *juniperoideum* were deposited in the DNA Data Bank of Japan Sequence Read Archive under the BioProject accession numbers PRJDB39622 and PRJDB39623, respectively.

## Supporting information

This article contains supporting information (22, 27).

## Conflict of interest

The authors declare that they have no conflicts of interest with the contents of this article.
