# Supplementary material for: Structural diversity of isoprene synthases in mosses from multiple terpenoid synthase lineages
Source: J Biol Chem. 2026 Mar 26;302(5):111410. doi: 10.1016/j.jbc.2026.111410 (PMC13098426; doi:10.1016/j.jbc.2026.111410)
Supplement: Supplementary material [file mmc1.docx]

**Supporting Information for**

Structural diversity of isoprene synthases in mosses from multiple terpenoid synthase lineages

Tetsuya Kawakami, Sho Miyazaki, Yuya Inoue and Hiroshi Kawaide

corresponding author: Hiroshi Kawaide

Email: hkawaide@cc.tuat.ac.jp

**This PDF file includes:**

Figures S1 to S10

Tables S1 to S6

Figures


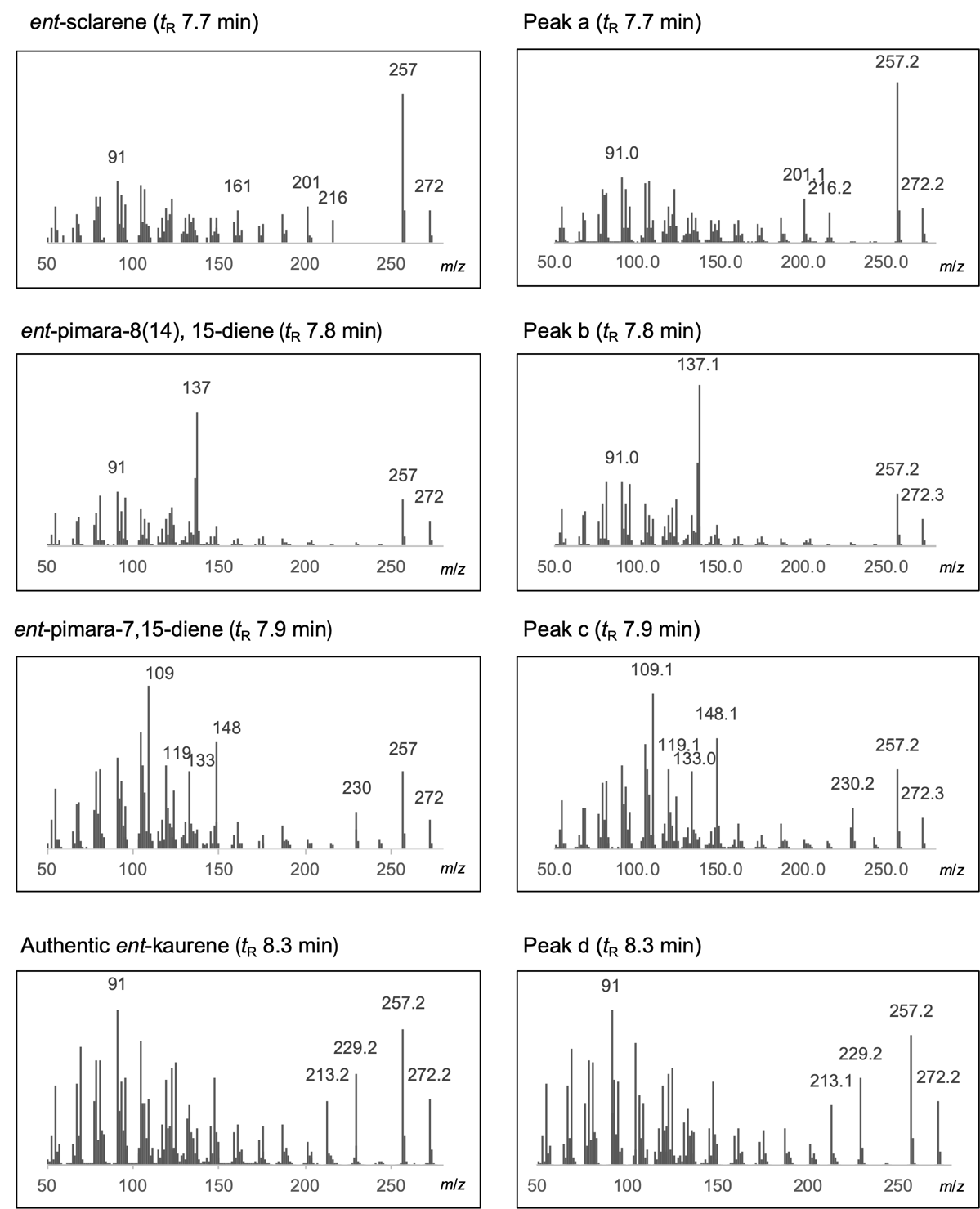


**Fig. S1.** Mass spectra of diterpenes produced by RcKSL2 and CpISPS mutants

Mass spectra of *ent*-sclarene, *ent*-pimara-8(14),15-diene, and *ent*-pimara-7,15-diene produced from RcKSL2:L633S/I636T, and peaks a–c from CpISPS:Y393A/F615A mutant. Mass spectra of *ent*-kaurene and peak d from CpISPS:Y393A/F615A/S502A/T505P.


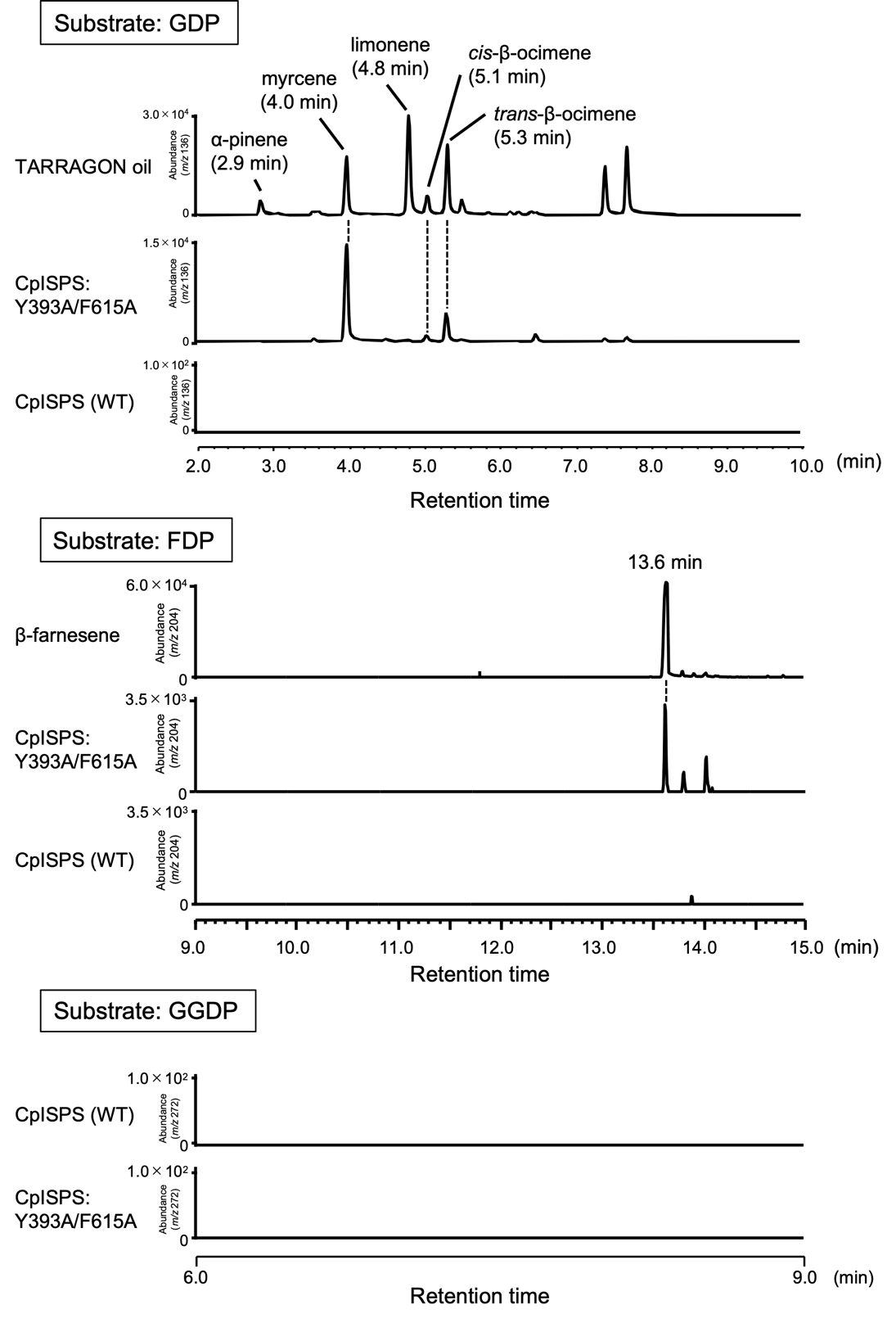


**Fig. S2.** Enzyme assay of CpISPS mutants with GDP, FDP and GGDP.


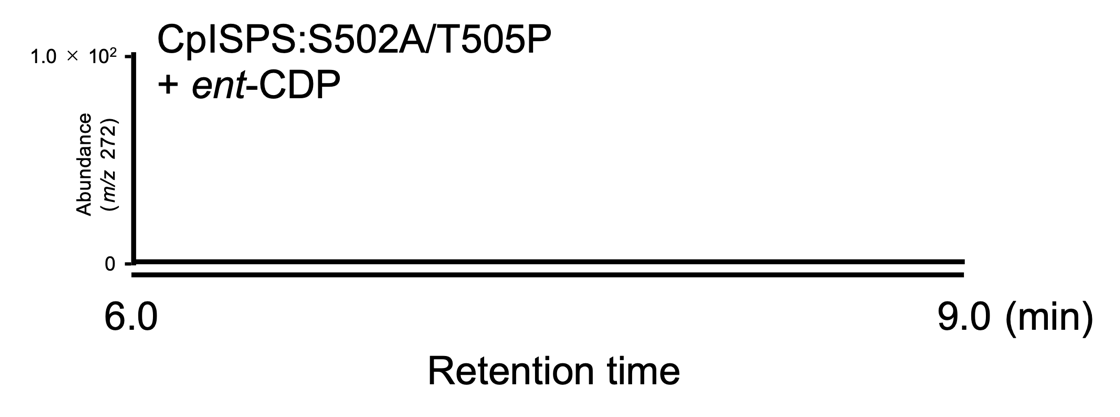


**Fig. S3.** Enzyme assay of CpISPS:S502A/T505P reacted with *ent*-CDP.


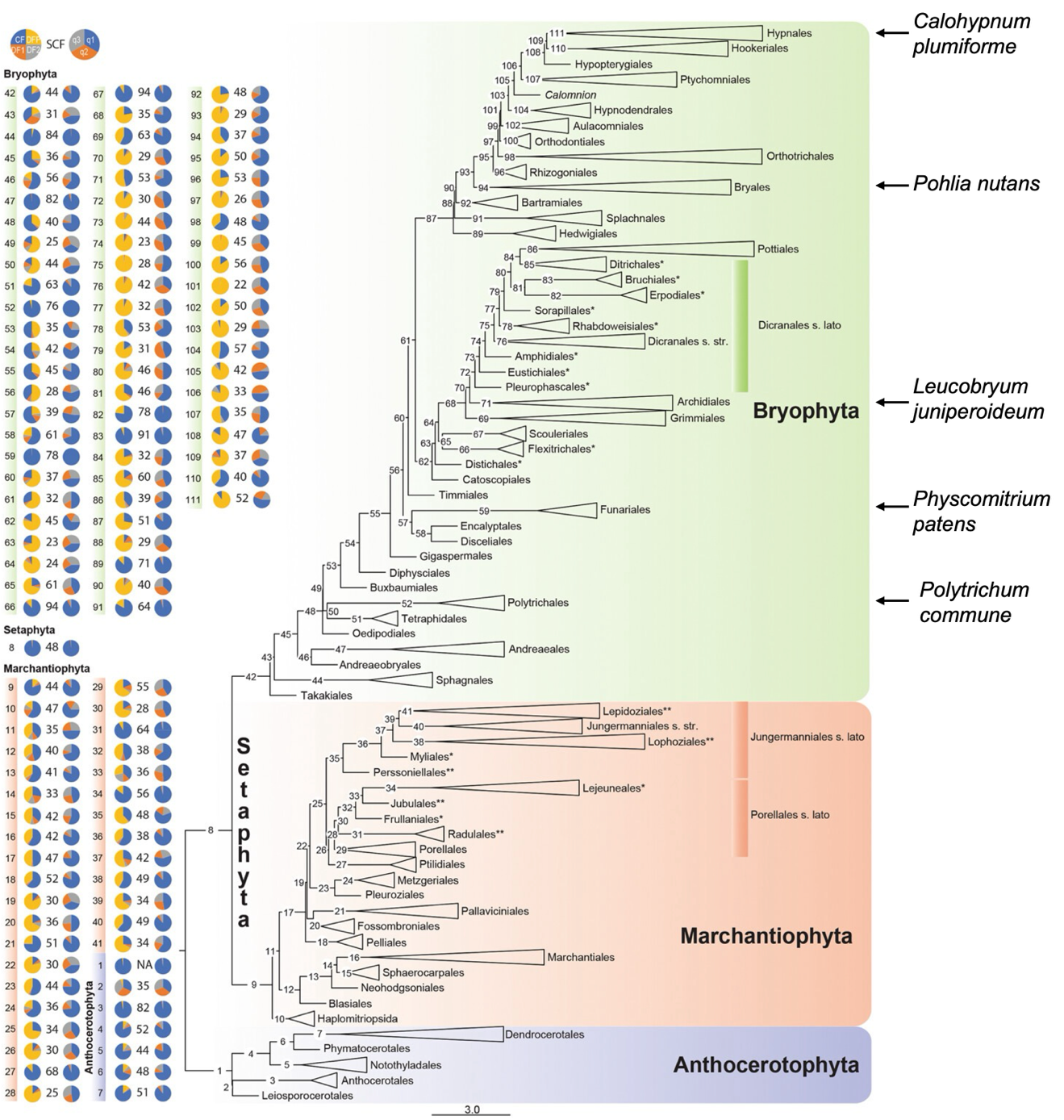


**Fig. S4.** Taxonomic orders of mosses and selected species for experiments.

Phylogenetic tree reproduced from Bechteler *et al*, under a CC BY-NC-ND license (27). Arrows indicate the phylogenetic position of mosses investigated in the present and previous study (22): *Calohypnum plumiforme* (Hypnales), *Pohlia nutans* (Bryales), *Leucobryum juniperoideum* (Archidiales), *Physcomitrium patens* (Funariales), *Polytrichum commune* (Polytrichales).


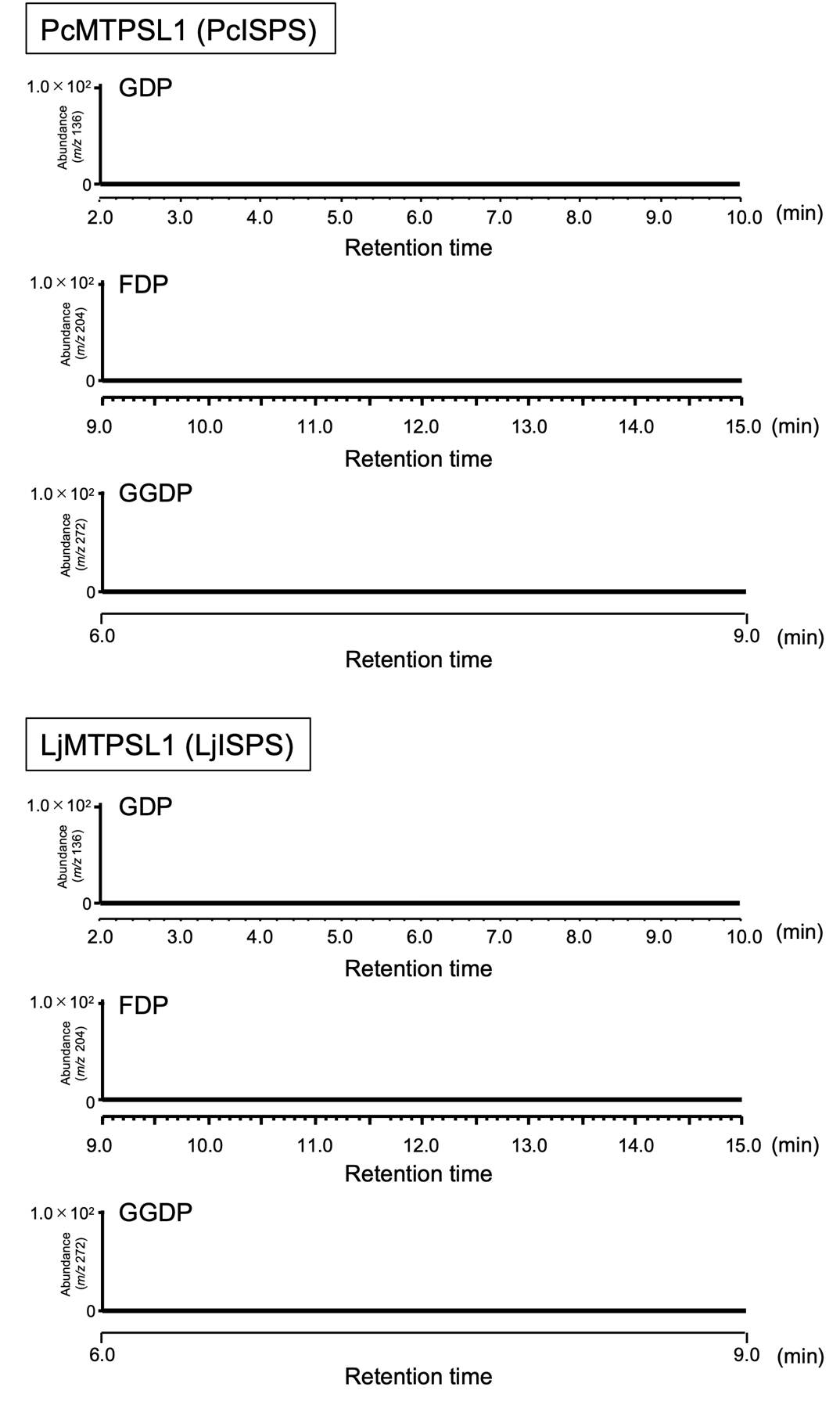


**Fig. S5.** Enzyme assay of MTPSL-type ISPSs with GDP, FDP and GGDP.


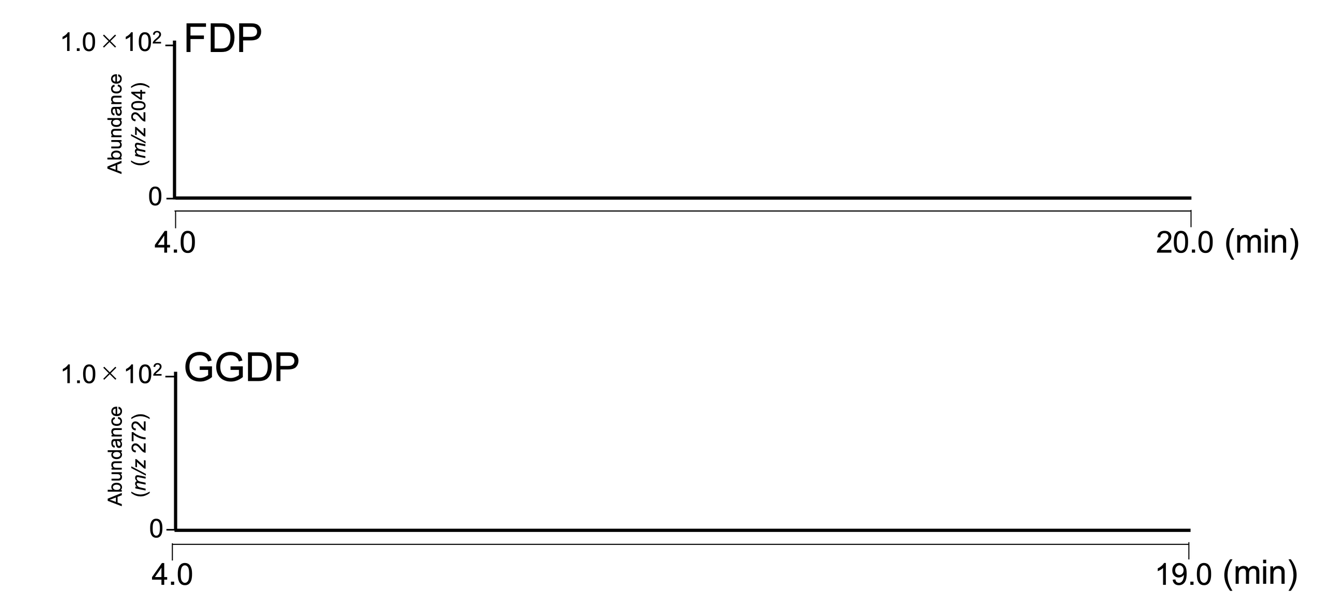


**Fig. S6.** Enzyme assay of PcISPS mutant with FDP and GGDP.


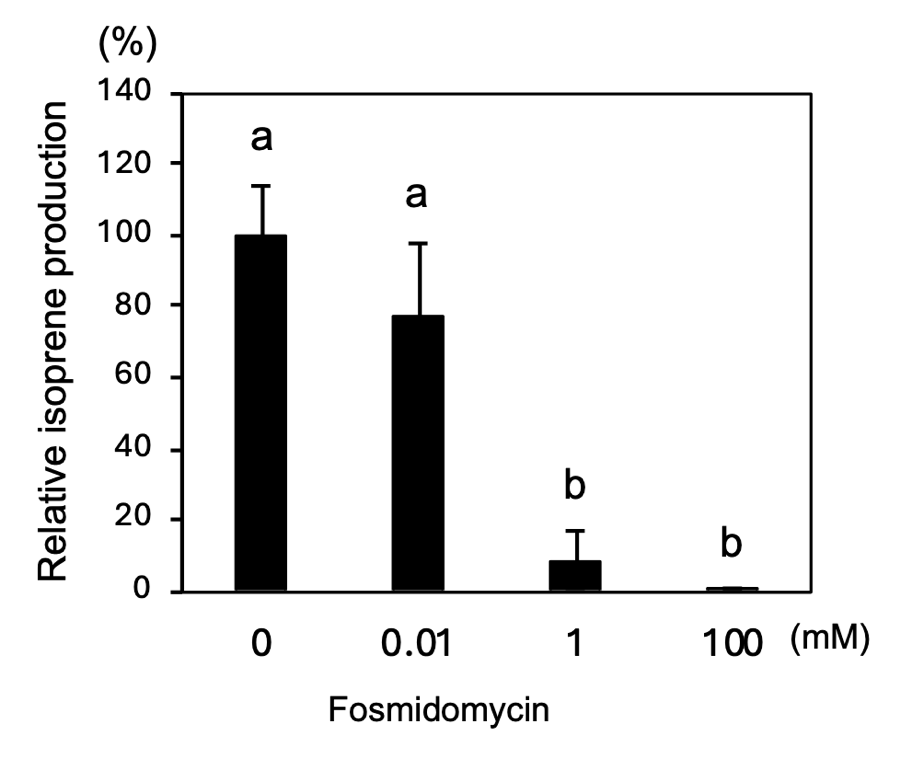


**Fig. S7.** Inhibitor assay for isoprene synthesis in *P. commune.*

The graph of the isoprene production in *P. commune* treated with fosmidomycin, which is an inhibitor of the MEP pathway operated in plastids. The values for each treatment group are presented as relative to the average isoprene production in the untreated control. The Tukey–Kramer test was performed following one-way ANOVA for statistical analysis (*n* = 3; F(3, 8) = 42.35, *p* < 0.01). Different letters indicate significant differences, with exact p-values provided in Table S3.


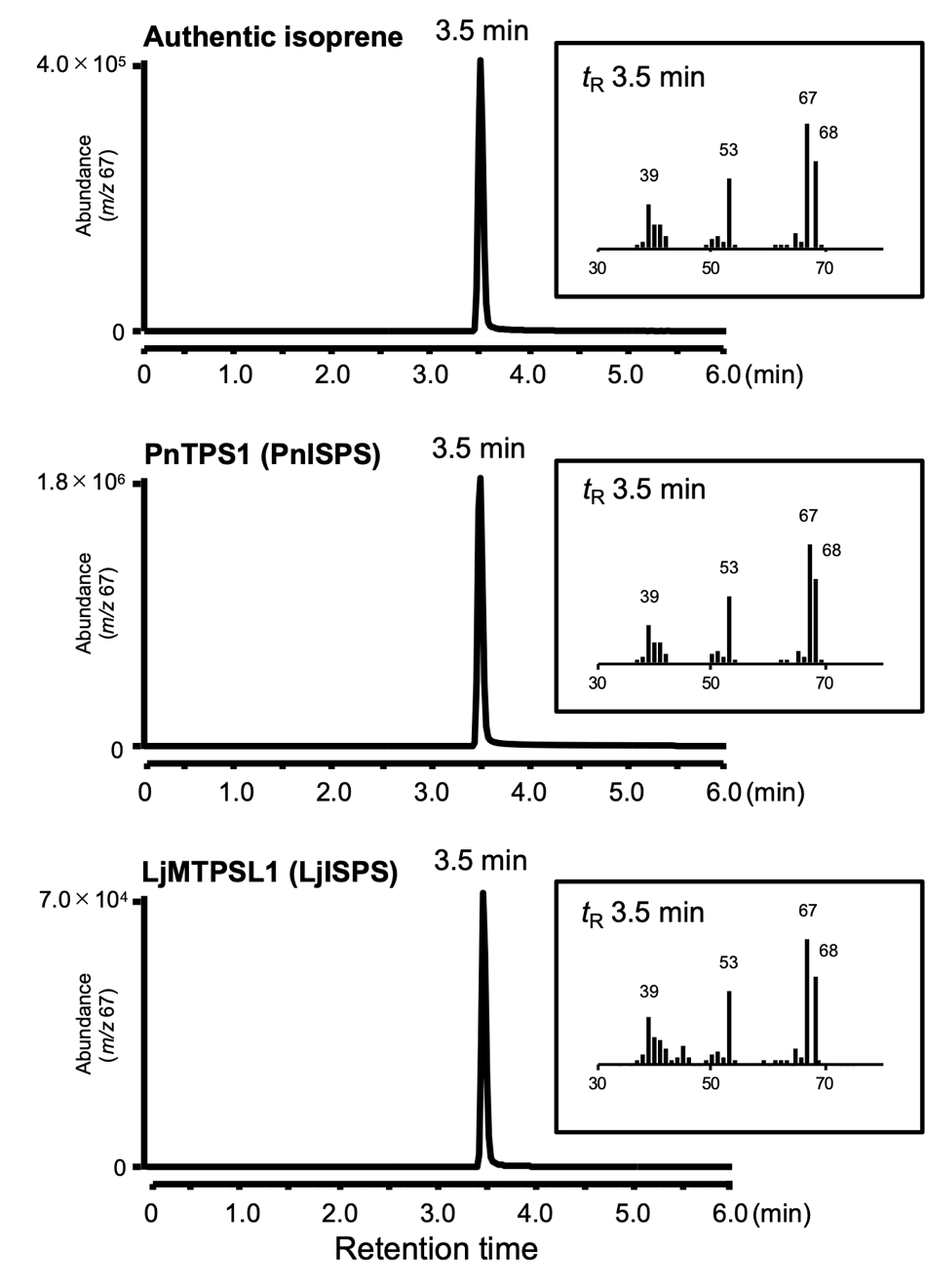


**Fig. S8.** Enzyme assays of PnISPS and LjISPS reacted with DMADP.


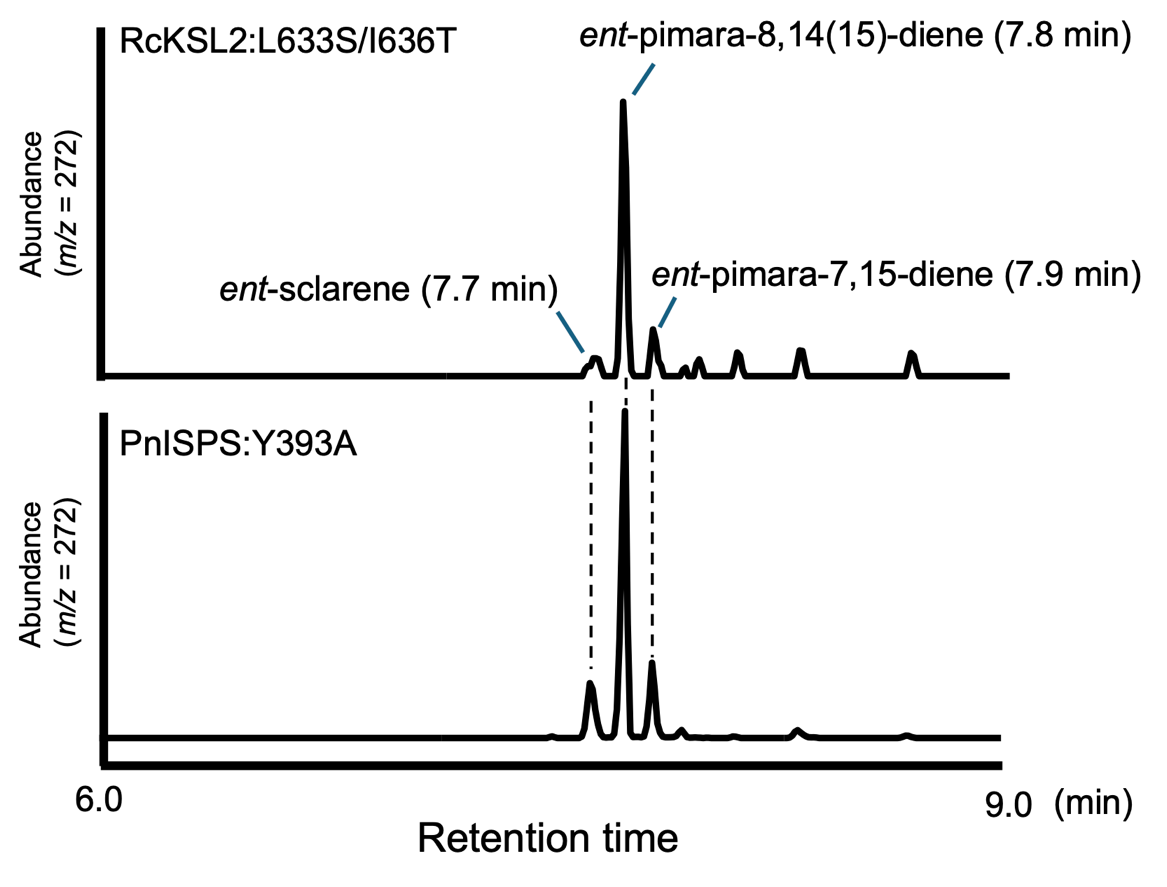


**Fig. S9.** Enzyme assays of cavity-expanded PnISPS mutants with *ent-*CDP.


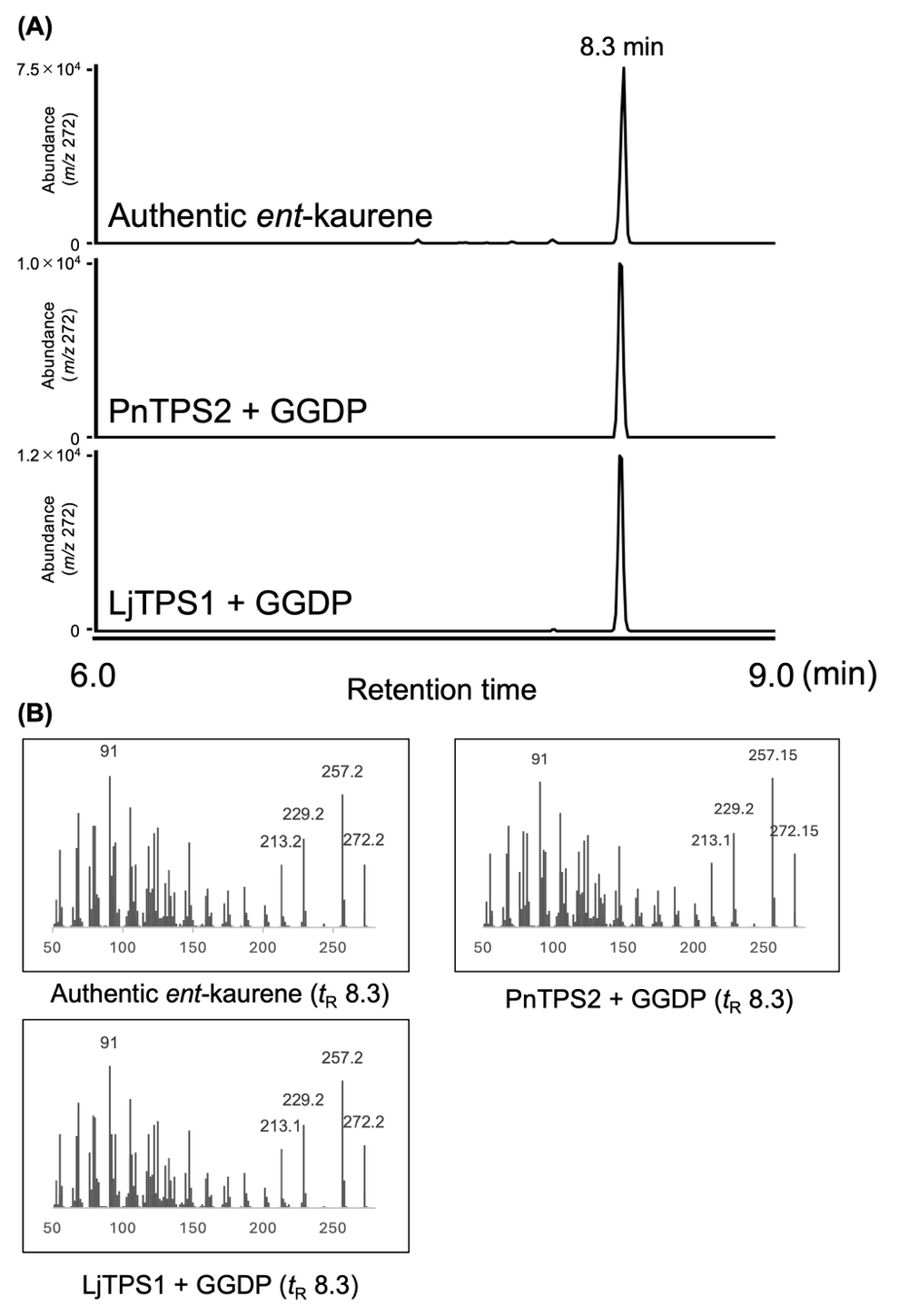


**Fig. S10.** Enzyme assays of bifunctional CPS/KSs from *P. nutans* (PnTPS2) and *L*. *juniperoideum* (LjTPS1) for *ent*-kaurene production. (A) Chromatograms from GC–MS analysis of authentic *ent*-kaurene standard, PnTPS2 with GGDP and LjTPS1 with GGDP. (B) Mass spectra of *ent*-kaurene standard, and mass spectra of each product from PnTPS2 and LjTPS1.

**Table S1.** The predicted volume of the active sites in TPSs.

**Table S2.** Primer Information for cDNA cloning and site-directed mutagenesis.

**Table S3.** Exact p-values for pairwise comparisons following one-way ANOVA in the inhibitor assay of Fig. S7

| **Fosmidomycin** | **0 mM** | **0.01 mM** | **1 mM** | **100 mM** |
| --- | --- | --- | --- | --- |
| **0 mM** | − | *p* = 0.22 | *p* < 0.01 | *p* < 0.01 |
| **0.01 mM** |  | − | *p* < 0.01 | *p* < 0.01 |
| **1 mM** |  |  | − | *p* = 0.86 |
| **100 mM** |  |  |  | − |

**Table S4.** The Reference genes for qPCR experiments in this study.

**Table S5.** Primer information for RT-PCR experiments of each ISPS gene.

**Table S6.** List of TPS and MTPSL genes used for phylogenetic tree analysis.
